# Algorithmic anxiety: AI, work, and the evolving psychological contract in digital discourse

**DOI:** 10.3389/fpsyg.2026.1745164

**Published:** 2026-02-17

**Authors:** Anurag Shekhar, Musawenkosi D. Saurombe

**Affiliations:** Department of Industrial Psychology and People Management, College of Business and Economics, University of Johannesburg, Auckland Park, South Africa

**Keywords:** algorithmic anxiety, artificial intelligence, conservation of resources, digital discourse, mixed methods, psychological contract, Reddit, self-determination theory

## Abstract

**Introduction:**

The study used a mixed-methods approach, utilising 1,454 Reddit narratives about AI-driven job displacement, to examine how AI is transforming the workplace psychological contract.

**Methods:**

This study used both quantitative and qualitative methods of analysis. It analysed sentiment patterns, emotional responses, and thematic content from digital discourse.

**Results:**

While our results show a surface level of optimism regarding the use of AI (52% of all sentiment was positive according to VADER), our results also showed a significant amount of negative sentiment (51% of all sentiment was negative according to BERT) that indicates a deeper concern of people in terms of their feelings of “algorithmic anxiety” related to job loss. Network analysis showed three interconnected discourse groups centered on employment disruption, ethical concerns, and technical systems (modularity Q = 0.42). Furthermore, seven themes emerged from the data analysis: shattered trust and corporate betrayal, eroded identities, technostress, devalued expertise, anxiety about the future, cynicism about adapting, and affirming human values, which illustrate how the use of AI has disrupted the psychological contract between employees and employers.

**Discussion:**

This study adds to psychological contract theory by illustrating ways that technology can breach an individual’s psychological contract at work. In addition, this study extends existing technostress literature by identifying specific sources of stress associated with AI use in the workplace. Finally, it applies self-determination theory to work settings where algorithms are shaping the work environment. Practically speaking, the findings suggest that employers who wish to address the growing problem of “algorithmic anxiety” should engage in transparent communication, involve employees in decision-making, and design their technological systems to preserve employee dignity in increasingly automated workplaces.

## 1 Introduction

The integration of artificial intelligence into workplaces represents not merely technological advancement but a fundamental reconfiguration of work itself, challenging centuries-old assumptions about human labor, expertise, and organizational relationships (Cramarenco et al., 2023; Murire, 2024). As organizations deploy increasingly sophisticated AI systems (from machine learning algorithms that make hiring decisions to autonomous agents that perform complex analytical tasks), the implicit social contracts binding employers and employees undergo unprecedented strain (Bankins et al., 2024; Murire, 2024; Soulami et al., 2024). This transformation is most visible in digital discourse where workers collectively process their experiences of displacement and resistance through online platforms that offer both anonymity and community (Dang and Liu, 2025; Gagné et al., 2022).

The integration of artificial intelligence into workplaces is profoundly uneven across geographic and economic contexts. In advanced economies like Germany, AI adoption has proceeded cautiously, with strong data protection frameworks and established worker consultation mechanisms shaping implementation (Bitkom, 2020; Özkiziltan and Hassel, 2021). However, even in these regulated environments, AI redistributes demand toward highly skilled professionals while creating “winners and losers” among vulnerable groups including women and older workers (Özkiziltan and Hassel, 2021). In emerging economies, digital disparities are further compounded by infrastructure gaps and limited technological access, determining whether AI serves as a catalyst for prosperity or a driver of economic exclusion (Calugan et al., 2025). The growth of platform-based gig work has introduced additional complexity, creating a “gray zone” between internal and external labor where workers face precarious employment managed by opaque, algorithmic systems (Keegan and Meijerink, 2025).

These structural disparities intersect with cultural factors that shape how workers experience AI-driven changes. Cultural orientations influence whether job displacement is processed through collectivist interdependent networks emphasizing social harmony or through individualist frameworks focusing on self-directed resource preservation (Hobfoll et al., 2018). AI’s pervasive presence fundamentally alters psychological contracts by reshaping unwritten expectations between employers and employees, often diminishing perceptions of autonomy, dignity, and job security (Moghayedi et al., 2024; Tomprou and Lee, 2022). The implementation of algorithmic management can trigger experiences of dehumanization, leaving workers feeling reduced to data points while experiencing heightened loneliness, cynicism, and technostress (Dang and Liu, 2025; Tarafdar et al., 2019).

The current moment represents a critical juncture in the history of work. Unlike previous waves of automation that primarily displaced manual labor, AI threatens knowledge work, creative professions, and roles previously considered uniquely human. Recent evidence suggests AI’s workplace impact is profoundly ambivalent and unevenly distributed (Cramarenco et al., 2023; Gagné et al., 2022; Oyekunle et al., 2024; Taslim et al., 2025; Zirar et al., 2023). While automation promises efficiency gains, cost reduction, and liberation from routine tasks, it simultaneously generates job insecurity, identity erosion, and novel forms of workplace stress that existing organizational frameworks struggle to address (Cramarenco et al., 2023; Soulami et al., 2024). The COVID-19 pandemic dramatically accelerated these dynamics, compressing years of anticipated digital transformation into months while leaving workers anxious about their continued relevance amid increasingly “intelligent” machines (Bankins et al., 2024; García-Madurga et al., 2024).

This acceleration has revealed profound gaps between technological capability and human adaptation (Frank et al., 2019; Johnson et al., 2020; Zirar et al., 2023). Organizations often implement AI with minimal consideration of psychological impacts, treating workforce transformation as a technical problem rather than a human one (Johnson et al., 2020; Zirar et al., 2023). Workers report feeling blindsided by automation decisions, betrayed by employers who promised job security, and increasingly uncertain about the sustainability of any career path (Bankins et al., 2024; Duggan et al., 2022). These experiences coalesce into what we term “algorithmic anxiety,” a complex syndrome encompassing not just fear of job loss but deeper concerns about human value, professional identity, and the meaning of work in an automated future (Dang and Liu, 2025; Kinowska and Sienkiewicz, 2023; Soulami et al., 2024).

Digital platforms, particularly Reddit, have emerged as crucial spaces where workers process these transformations (Amaya et al., 2021; De Choudhury and De, 2014). Reddit’s structure, combining anonymity with community validation through upvoting creates unique conditions for authentic disclosure (Amaya et al., 2021; De Choudhury and De, 2014; Kahlow, 2024). Workers share experiences too sensitive for workplace discussion, collectively constructing narratives about AI’s impact that often contradict official corporate communications (Andalibi et al., 2018; De Choudhury and De, 2014; Nukhu et al., 2025). These digital narratives reveal not just individual distress but emergent patterns of collective sense-making about technology, work, and human value in the algorithmic age (Boyd and Crawford, 2012; De Choudhury and De, 2014; Leavitt and Robinson, 2017; Nukhu et al., 2025).

The significance of understanding these dynamics extends far beyond documenting worker experiences. As AI capabilities expand toward artificial general intelligence, the question is not whether human work will be transformed but how society will manage that transformation (Calugan et al., 2025; Khogali and Mekid, 2023; Zirar et al., 2023). Current approaches often prioritize technical efficiency and economic optimization while neglecting human considerations, leading to resistance, disengagement, and the erosion of organizational trust (Calugan et al., 2025; Kinowska and Sienkiewicz, 2023; Leicht-Deobald et al., 2019; Ragu-Nathan et al., 2008). Without understanding the psychological and social dimensions of AI integration, organizations risk undermining the very productivity gains they seek while inflicting unnecessary human suffering.

Despite growing research attention, significant knowledge gaps persist in understanding AI’s psychosocial workplace impacts (Dang and Liu, 2025; Kinowska and Sienkiewicz, 2023; Soulami et al., 2024; Vrontis et al., 2022). Existing studies predominantly focus on narrow contexts with limited attention to the lived experiences of those directly displaced by automation (Bankins et al., 2024; Khogali and Mekid, 2023; Soulami et al., 2024; Zirar et al., 2023). Methodological approaches remain fragmented, with quantitative studies often lacking emotional nuance, while qualitative research often lacks the scale to identify broader patterns (Köchling and Wehner, 2020; Soulami et al., 2024). Few studies successfully integrate computational and qualitative methods to capture both the breadth and depth of worker experiences in the face of algorithmic disruption (Taslim et al., 2025).

Most critically, theoretical frameworks for understanding technology-mediated psychological contracts remain underdeveloped (Bankins et al., 2024; Keegan and Meijerink, 2025). Traditional organizational theories assume human actors on both sides of the employment relationship, but what happens when algorithms make decisions previously reserved for human managers (Keegan and Meijerink, 2025)? How do workers maintain professional identity when machines perform their core tasks (Dang and Liu, 2025; Keegan and Meijerink, 2025; Khogali and Mekid, 2023)? What new forms of resistance emerge when traditional labor organizing confronts algorithmic management (Gagné et al., 2022; Golgeci et al., 2025; Keegan and Meijerink, 2025)? These questions require not just empirical investigation but theoretical innovation (Özkiziltan and Hassel, 2021).

This study addresses these gaps through a comprehensive mixed-methods analysis of Reddit discourse about AI-driven job displacement. We analyze 1,454 comments from a discussion thread explicitly soliciting narratives from workers affected by automation, combining computational text analytics to map discourse patterns with qualitative thematic analysis to understand meaning and context. The discourse community includes both workers directly displaced by AI and those experiencing anticipatory anxiety about potential future displacement, with community engagement through upvoting and commenting revealing collective sense-making processes. We apply four theoretical lenses (psychological contract theory, technostress, self-determination theory, and conservation of resources) to interpret how workers experience and collectively construct understandings of AI-driven workplace transformation. Our aim is to illuminate the human dimensions of algorithmic management, contributing both theoretical insights for scholars and practical guidance for organizations seeking to implement ethical AI.

## 2 Literature review

### 2.1 Theoretical foundations for understanding AI’S workplace impact

The psychological ramifications of AI in the workplace cannot be understood through a single theoretical lens. Instead, we must integrate multiple perspectives to capture the complexity of human responses to algorithmic disruption. Four theoretical frameworks prove particularly illuminating: psychological contract theory, technostress theory, self-determination theory, and conservation of resources theory. Each offers unique insights while together providing a comprehensive understanding of algorithmic anxiety.

Psychological contract theory (Rousseau, 1995) provides the foundational framework for understanding how AI disrupts workplace relationships. This theory posits that beyond formal employment contracts, workers and employers maintain implicit expectations of mutual obligations. Employees expect fair treatment, job security, and opportunities for career development in exchange for their loyalty, effort, and commitment. Recent empirical work reveals how AI fundamentally challenges these assumptions. When organizations implement AI systems that lead to layoffs or significant role changes, workers perceive not just strategic business decisions but profound violations of trust (Bankins et al., 2024; Soulami et al., 2024).

The nature of these violations differs qualitatively from traditional organizational changes. Kinowska and Sienkiewicz (2023) found that AI-driven decisions feel particularly impersonal and arbitrary, lacking the human element that traditionally cushioned difficult organizational transitions. Workers report feeling “*betrayed by an algorithm*,” a phrase that captures the unique sense of alienation that comes with being evaluated, managed, or replaced by non-human entities. The psychological contract (built on assumptions of human reciprocity) struggles to accommodate relationships where one party is algorithmic. This creates what Keegan and Meijerink (2025) term “algorithmic accountability gaps,” where workers cannot identify whom to hold responsible for AI-driven decisions affecting their livelihood.

Technostress theory (Tarafdar et al., 2019) highlights the psychological strain associated with technology adoption, with AI introducing stressors that are qualitatively different from those of traditional information systems. The framework identifies five technostress creators: techno-overload (excessive demands resulting from technology use), techno-invasion (technology blurring work-life boundaries), techno-complexity (the constant need to learn new systems), techno-insecurity (fear of being replaced by technology), and techno-uncertainty (continuous technological changes). AI amplifies each dimension while adding novel stressors unique to intelligent systems (Tarafdar et al., 2019).

Recent empirical studies demonstrate AI’s distinctive stress profile. Algorithmic management creates “performance anxiety loops” where workers feel perpetually monitored and evaluated by opaque systems they neither understand nor trust (García-Madurga et al., 2024; Segkouli et al., 2023). Unlike traditional IT that workers can master through training, AI systems continuously evolve, creating perpetual techno-complexity. Zirar et al. (2023) document how AI-related technostress involves existential dimensions absent from traditional technology stress; workers question not just their competence with tools but their fundamental value as humans. The concept of “algorithmic precarity” emerges, where workers experience chronic uncertainty about whether their skills, regardless of proficiency level, will remain relevant.

Self-determination theory (Ryan and Deci, 2000) identifies three fundamental psychological needs essential for well-being and motivation: autonomy, competence, and relatedness. Autonomy suffers when algorithmic management dictates work processes through rigid protocols that eliminate human judgment and creativity. Gagné et al. (2022) provide extensive evidence that algorithmic management reduces perceived autonomy as workers cannot negotiate with or influence algorithmic decisions.

Competence needs are threatened when AI systems outperform humans at tasks previously defining professional identity (Golgeci et al., 2025). Customer service workers report feeling isolated when AI handles initial client contact, leaving humans to manage only escalated problems (Johnson et al., 2020). The cumulative effect is profound demotivation, with workers questioning the purpose of developing skills that machines can instantly replicate (Gagné et al., 2022; Golgeci et al., 2025).

Conservation of resources theory (Hobfoll, 1989) frames AI anxiety as a response to a perceived threat or loss of resources. This theory posits that individuals strive to obtain, retain, and protect resources they value, whether material (such as salary and job security), personal (including skills and health), or social (including status and relationships) (Hobfoll et al., 2018; Leicht-Deobald et al., 2019). Stress occurs when resources are threatened, lost, or when investment fails to yield expected returns. AI threatens multiple resource categories simultaneously (Golgeci et al., 2025).

The threat begins with potential job loss (material resource) but quickly cascades (Hobfoll, 1989; Hobfoll et al., 2018; Özkiziltan and Hassel, 2021). Professional identity (a personal resource) erodes when machines perform tasks that define it. Social status diminishes when expertise becomes obsolete (Calugan et al., 2025; Hobfoll, 1989). Future planning capacity (psychological resource) suffers under radical uncertainty about career viability (Hobfoll, 1989; Tenakwah and Watson, 2025; Zirar et al., 2023). Even workers retaining their positions experience resource loss through “skill hollowing,” where AI handles challenging tasks, leaving humans with either mundane work or high-stress exception handling (Ashok et al., 2022; Hobfoll, 1989; Özkiziltan and Hassel, 2021). The anticipatory nature of these threats (i.e., workers fearing future automation) creates chronic stress that depletes coping resources before actual displacement occurs (Golgeci et al., 2025; Hobfoll et al., 2018).

### 2.2 Empirical evidence: the state of knowledge

Recent systematic reviews reveal AI’s profoundly ambivalent impact on the workplace. While AI-driven automation can boost efficiency and reduce monotonous work, it also increases job insecurity and the need for continuous reskilling (Cramarenco et al., 2023; Soulami et al., 2024). The impact is uneven across sectors and regions, with non-Western and lower-skilled workers facing greater risks (Cramarenco et al., 2023; Moghayedi et al., 2024; Soulami et al., 2024; Zirar et al., 2023).

The COVID-19 pandemic significantly altered the trajectories of AI adoption, particularly in the wake of accelerated digital transformation (Cramarenco et al., 2023; Khogali and Mekid, 2023; Soulami et al., 2024). Organizations implemented AI systems rapidly during the pandemic, often bypassing normal change management processes (Bankins et al., 2024; García-Madurga et al., 2024).

Organizational trust emerges consistently as a critical casualty of algorithmic management. AI-driven decision-making can erode organizational trust and psychological contracts, particularly when perceived as impersonal or leading to layoffs (Bankins et al., 2024; Kinowska and Sienkiewicz, 2023; Soulami et al., 2024). Employees report feelings of betrayal and reduced reciprocity, especially when algorithmic management lacks transparency or fairness (Kinowska and Sienkiewicz, 2023; Pereira et al., 2023; Taslim et al., 2025). The effects are more pronounced in gig work, call centers, and sectors with high algorithmic oversight (Kinowska and Sienkiewicz, 2023; Vrontis et al., 2022; Zirar et al., 2023).

### 2.3 Ethical dimensions and moral injury

The ethical implications of workplace AI extend beyond traditional concerns about bias and privacy to fundamental questions about human dignity and the nature of work itself. Ethical concerns (i.e., fairness, bias, privacy, and human dignity) are central to debates on AI in the workplace (Cheng et al., 2022; Hunkenschroer and Luetge, 2022; Kordzadeh and Ghasemaghaei, 2022; Oyekunle et al., 2024). Moral injury and alienation are reported when employees feel replaced or surveilled by AI (Dang and Liu, 2025; Gratch and Fast, 2022; Hunkenschroer and Luetge, 2022; Oyekunle et al., 2024).

Ethics and discrimination in artificial intelligence-enabled recruitment practices reveal systematic bias patterns (Chen, 2023). Algorithmic bias in recruitment and performance management can perpetuate discrimination and erode perceptions of fairness (Chen, 2023; Hunkenschroer and Luetge, 2022; Kordzadeh and Ghasemaghaei, 2022; Oyekunle et al., 2024; Starke et al., 2022).

Ethics of AI-Enabled Recruiting and Selection research identifies multiple categories of ethical harm from workplace AI (Hunkenschroer and Luetge, 2022). Workers experiencing AI-driven changes report risks associated with dehumanization due to artificial intelligence use (Dang and Liu, 2025). Algorithmic evaluation and AI-driven HR practices affect employees’ sense of competence, autonomy, and purpose, with creative professionals and knowledge workers particularly sensitive to perceived dehumanization (Bankins et al., 2024; Cheng et al., 2022; Gagné et al., 2022).

### 2.4 Digital discourse as a nexus for collective interpretation

Digital platforms afford researchers unprecedented opportunities to observe authentic employee experiences, often unavailable to conventional organizational studies (Brown et al., 2018). Traditional workplace research often encounters pervasive response bias because employees are reluctant to express negative opinions, fearing professional repercussions that could impact their employment status (Amaya et al., 2021; Boyd and Crawford, 2012). Platforms such as Reddit mitigate these limitations because their pseudonymous or anonymous nature facilitates candid discourse and low inhibition (Brown et al., 2018; Kahlow, 2024). This reliance on dissociative anonymity enables individuals to express intimate details they would otherwise withhold in contexts where they could be identified (Kahlow, 2024; Sit et al., 2024). Furthermore, community features, including voting and commentary, enable the collective validation and interpretation of shared experiences (Amaya et al., 2021; De Choudhury and De, 2014; Leavitt and Robinson, 2017).

Research confirms the distinctive value of platforms like Reddit for investigating sensitive or stigmatizing issues, particularly within mental health discourse (De Choudhury and De, 2014; Kahlow, 2024). Studies demonstrate that Reddit’s architecture, which combines the ability to employ varying degrees of anonymity (including “throwaway” accounts) with community curation via voting, generates exceptionally rich data about challenging topics (De Choudhury and De, 2014). This environment encourages users toward deeper self-disclosure, enabling narratives related to mental illness, work, and personal relationships that would typically be too sensitive or risky in identifiable settings (De Choudhury and De, 2014; Kahlow, 2024; Sit et al., 2024).

Nevertheless, the systematic analysis of massive digital discourse introduces inherent methodological difficulties (Boyd and Crawford, 2012; Proferes et al., 2021). Automated tools like sentiment analysis and Natural Language Processing (NLP) frequently struggle with the complexities inherent in social communication (Balcioğlu et al., 2025). The interpretation of context in digital environments is problematic, as nuances such as sarcasm, irony, cultural references, and typos can severely confound sophisticated algorithms seeking to interpret meaning (Balcioğlu et al., 2025). Researchers must actively address this complexity, recognizing that data interpretation is inherently subjective and that claims of objectivity are often misleading, particularly when information is removed from its original conversational context (Boyd and Crawford, 2012). A sophisticated contextual analysis framework is necessary to isolate genuine sentiment from broader community trends and account for temporal biases, thereby improving the reliability of the findings (Balcioğlu et al., 2025).

## 3 Methods

### 3.1 Research design and epistemological framework

This study employs an explanatory sequential mixed-methods design (Creswell and Clark, 2017) to examine AI’s psychosocial workplace impacts through digital discourse analysis. This approach integrates computational text analytics for pattern identification with qualitative thematic analysis for meaning interpretation. The sequential design allows quantitative findings to inform qualitative investigation, while maintaining flexibility for emergent insights.

Our epistemological stance combines critical realism with social constructivism, and this dual perspective directly shaped our analytical approach at multiple levels. Critical realism proposes that reality exists independently of our perceptions but is only accessible through socially mediated interpretation (Bhaskar, 1975). Social constructivism emphasizes that meaning emerges through collective sense-making processes (Berger and Luckmann, 1966). These seemingly contradictory positions prove complementary when analyzing workplace AI discourse.

We operationalized this epistemological framework through specific analytical decisions. First, we treat certain elements as objectively real (the ontological dimension): Retrenchment at work, wage reductions, and organizational restructuring described by participants are taken as factual events that occurred in the material world. Our computational analysis of discourse patterns (sentiment distributions, topic frequencies, network structures) similarly treats the text corpus as an objective reality amenable to systematic measurement.

Second, we simultaneously treat the meaning and emotional significance of these events as socially constructed (the epistemological dimension). Workers collectively construct interpretations of AI’s impact through digital discourse, negotiating whether displacement represents “innovation,” “betrayal,” “inevitability,” or “injustice.” Our qualitative thematic analysis examines this meaning-making process, recognizing that “algorithmic anxiety” is not a direct physiological response to technology but a culturally and linguistically mediated psychological experience. The same objective event (being replaced by AI) can be experienced and narrated differently depending on interpretive frameworks available within particular discourse communities.

Third, we recognize that digital discourse simultaneously reflects and constitutes reality (the dialectical relationship). Reddit comments both represent workers’ existing psychological states and actively construct those states through the act of articulation and community validation. When a worker writes “I feel betrayed by my employer” and receives upvotes and supportive comments, the psychological experience of betrayal is both expressed and intensified through the discursive process. This reflexive quality justifies our attention to discourse structure (how meaning is collectively constructed) alongside discourse content (what is being experienced).

This epistemological stance justified our methodological integration: computational methods capture patterns in the objective reality of discourse structure (what is observable and measurable), while qualitative interpretation examines how workers collectively construct meaning from those patterns (what significance they assign to events). The divergence between VADER and BERT sentiment analysis exemplifies this approach—we treat both the surface linguistic features (objective) and the contextual meaning (constructed through linguistic conventions about irony and sarcasm) as simultaneously real and worthy of analysis.

### 3.2 Data collection and sampling

We analyzed 1,454 comments from the Reddit thread “*Hey people who lost their jobs to AI, what happened?*” posted on r/AskReddit in 2025. This thread generated exceptional engagement (over 5,000 upvotes), providing one of the largest collections of firsthand accounts about AI displacement available for research. The r/AskReddit community, with 35 million members, represents diverse demographics and occupations, offering broader perspective than profession-specific forums.

Data collection was conducted through Reddit’s official API, ensuring the complete capture of public comments while respecting the platform’s terms of service. We excluded comments that were deleted, moderator posts, and bot-generated content. Comments ranged from brief responses (five words) to detailed narratives (over 2,000 words), with a median length of 73 words. The temporal concentration (most comments posted within 72 h) captures a synchronous collective discussion rather than scattered individual posts.

The thread’s framing *(“Hey people who lost their jobs to AI, what happened?”)* introduces systematic selection bias toward negative experiences of AI-driven workplace transformation. This framing explicitly solicits displacement narratives, attracting workers who experienced AI implementation as threatening, disruptive, or unjust, while likely excluding those whose AI experiences were neutral, positive, or genuinely augmenting. However, the discourse community engaging with these narratives extends beyond those directly displaced. The upvoting system and comment threads reveal participation from workers experiencing anticipatory anxiety about potential future displacement, sympathetic observers witnessing AI’s impact on colleagues or industries, and knowledge workers more broadly recognizing themselves in these narratives. This broader engagement pattern suggests the discourse captures not only direct experiences of displacement but also collective sense-making about AI’s implications across the knowledge work sector. The most highly upvoted comments (some receiving 10,000 + upvotes) indicate widespread resonance with displacement narratives, suggesting these experiences express concerns shared by workers not yet directly affected but recognizing their potential future in others’ present circumstances.

We contend this sampling characteristic shapes the scope of our findings while also providing methodological value aligned with our research objectives. Rather than seeking to measure the prevalence of different AI experiences across the working population, we aimed to understand the phenomenology of algorithmic anxiety—both the lived experience of workers directly displaced and the anticipatory anxiety of those witnessing displacement and recognizing their vulnerability. The selection bias toward negative experiences provides access to rich, authentic narratives from those directly impacted, while the community validation through upvotes and supportive comments reveals how these narratives resonate with broader worker populations experiencing vicarious or anticipatory anxiety. This engagement pattern illuminates how algorithmic anxiety functions as both individual psychological response and collective social phenomenon.

The comments reveal diverse experiences within this negatively-affected population, including complete job elimination, partial role automation, forced early retirement, and pre-emptive career changes. Industries represented include creative services, data analysis, customer support, manufacturing, legal services, and education, suggesting the phenomenon crosses occupational boundaries. However, our findings characterize the experience and collective construction of algorithmic anxiety among workers directly or vicariously affected, rather than estimating the prevalence of such experiences in the general working population. Claims about how commonly workers experience AI negatively, what proportion experience algorithmic anxiety, or whether negative outcomes are inevitable would be unwarranted extrapolations from our data. The interpretive consequences of this sampling approach are discussed further in the Limitations section.

### 3.3 Computational analysis pipeline

We implemented six complementary computational techniques to map discourse structure and emotional patterns. These methods were selected to capture different facets of emotional expression that single approaches might miss. VADER and BERT were paired deliberately to examine whether surface language aligns with contextual meaning in discourse about job displacement. VADER represents widely-used lexicon-based approaches optimized for social media (Hutto and Gilbert, 2014), while BERT’s transformer architecture captures contextual complexity and has demonstrated advantages on texts containing sarcasm (Ribeiro et al., 2016; Saha et al., 2022). The NRC Emotion Lexicon allows examination of discrete emotions beyond polarity (Mohammad and Turney, 2013), while LDA identifies thematic structure without researcher preconception (Blei, 2012). Network analysis reveals how concepts cluster in workers’ thinking. This multi-method approach provides complementary insights into the corpus.

Sentiment analysis comparison: We employed two distinct approaches to understand the complexity of emotional valence. VADER (Valence Aware Dictionary and sEntiment Reasoner), a rule-based tool optimized for social media, calculates sentiment through lexicon matching with adjustments for intensifiers, negations, and punctuation. Each comment received four scores: positive, negative, neutral, and compound (normalized aggregate). Classification thresholds followed standard conventions: compound scores ≥ 0.05 indicated positive sentiment, <−0.05 indicated negative sentiment, and intermediate values indicated neutral sentiment.

For contextual analysis, we employed RoBERTa (Robustly Optimized BERT Pretraining Approach), fine-tuned on Twitter data. This transformer model uses attention mechanisms to consider word relationships across entire comments, better capturing sarcasm, irony, and contextual meaning. The model processed comments in 32-item batches with a maximum length of 512 tokens, outputting categorical predictions along with confidence scores.

The divergence between models (52% positive for VADER versus 51% negative for BERT) warrants explanation, as it reveals important differences in how these tools process emotionally complex text. Comparative studies indicate that lexicon-based methods like VADER and transformer-based approaches like BERT capture different dimensions of sentiment, with BERT demonstrating advantages on datasets containing sarcasm and contextual complexity (Ribeiro et al., 2016; Saha et al., 2022). VADER operates by matching words against pre-scored sentiment dictionaries, treating terms like “free,” “opportunity,” and “great” as inherently positive regardless of context (Hutto and Gilbert, 2014). BERT’s transformer architecture examines word relationships across entire sentences through attention mechanisms, allowing it to capture how surrounding context modifies apparent sentiment (Devlin et al., 2019).

Manual inspection of the 659 comments where models disagreed most strongly revealed patterns suggesting VADER misclassified resigned acceptance and defensive humor as genuine positivity. Comments like “Great news, everyone gets to retrain for jobs that do not exist yet!” scored positive in VADER (detecting “great” and “news”) but negative in BERT (recognizing sarcasm through contextual cues). Similarly, “At least I have more time to update my resume daily” triggered positive VADER scores (detecting “more time”) while BERT identified underlying negativity through implied futility. These patterns echo findings from pandemic-related discourse, where VADER misclassified anxiety-laden posts based on surface markers while contextual models detected underlying distress (Saha et al., 2022).

For this study, we treat BERT’s contextual analysis as the more appropriate measure of underlying sentiment for our corpus, while recognizing VADER’s results as potentially revealing how workers present their experiences in public forums. This interpretation assumes that ironic and resigned language reflects genuine negative emotion rather than neutral coping, an assumption we acknowledge may not hold universally. The discordance between methods informed our qualitative coding by directing attention to ironic expressions, resigned acceptance, and gallows humor as potentially significant features of the discourse rather than dismissing them as noise. However, this interpretation cannot be definitively validated with our current data. Alternative explanations remain plausible: workers might experience genuine emotional ambivalence rather than masked negativity, humor might represent effective coping that genuinely reduces distress rather than concealing it, or the divergence might partly reflect model-specific limitations rather than solely revealing emotional complexity. Validation would require complementary methods unavailable in our design (such as physiological stress measures, behavioral observations, or follow-up interviews with participants) to establish whether BERT’s classifications better approximate participants’ actual emotional states than VADER’s classifications.

Discrete emotion analysis: The NRC Emotion Lexicon mapped eight basic emotions (anger, fear, anticipation, trust, surprise, sadness, joy, disgust) plus positive/negative affect. NRC lexicon was selected over alternatives because its crowdsourced development provides coverage of the eight basic emotions identified in psychological research (Mohammad and Turney, 2013), offering finer-grained emotional mapping than simple polarity measures. This allows examination of whether workers experience complex emotional mixtures (e.g., simultaneous trust and fear) rather than uniform negative or positive states. This word-association approach counts emotion-linked terms, providing granular emotional mapping beyond simple polarity. Results showed complex emotional co-occurrence: trust (13%) appeared alongside fear (6%), suggesting simultaneous faith in technology and personal anxiety.

Topic modeling for thematic discovery: Latent Dirichlet Allocation with eight topics optimally balanced interpretability with distinctiveness. The algorithm assumes documents contain mixtures of topics, with topics defined by word probability distributions. LDA was selected because its probabilistic approach allows comments to contain mixtures of topics, reflecting how workers may simultaneously discuss practical, emotional, and ethical dimensions rather than discrete separable themes (Blei, 2012). The eight-topic solution was determined through coherence score optimization, balancing interpretability with distinctiveness. Hyperparameter tuning (*α* = 0.1, *β* = 0.01) encouraged sparse topic assignment. Coherence scores validated topic quality (C_v = 0.52), indicating meaningful semantic clusters.

Keyword Significance Testing: TF-IDF (Term Frequency-Inverse Document Frequency) analysis identified statistically distinctive vocabulary. This technique weighs word importance by frequency within documents against rarity across the corpus. We retained the top 50 terms after removing stop words and applying frequency thresholds (minimum 5 occurrences maximum 80% document frequency).

Semantic network construction: Co-occurrence networks visualized term relationships, with edges weighted by within-comment co-appearance frequency. This graph-based approach complements topic modeling by revealing which concepts cluster together in workers’ thinking, exposing cognitive associations that may not emerge as discrete topics. Network structure can indicate whether discourse is fragmented or integrated across different concerns. We applied modularity optimization (Louvain algorithm) for community detection, identifying cohesive term clusters. The resulting network showed three primary communities with modularity Q = 0.42, indicating meaningful structural divisions.

Statistical validation: Inter-method reliability assessed through convergent validity testing. Sentiment classifications showed fair agreement (Cohen’s *κ* = 0.24), while topic assignments demonstrated moderate stability across multiple runs (average Jaccard similarity = 0.68).

### 3.4 Qualitative analysis protocol

Thematic analysis followed Braun and Clarke's (2006) reflexive approach, emphasizing researcher interpretation rather than theme “discovery.” We utilized Atlas.ti for data management and coding.

Coding framework development: We created seven intentional coding questions bridging computational findings with theoretical frameworks:

1. How do individuals emotionally respond to AI-driven change? (linking to sentiment/emotion findings)
2. How do commenters describe organizational fairness and trust? (connecting to psychological contract theory)
3. How has AI impacted professional identity and meaning? (relating to self-determination theory)
4. What technology-induced stresses do workers experience? (examining technostress dimensions)
5. How do individuals adapt to or resist AI adoption? (exploring agency and coping)
6. How do workers connect personal experiences to societal patterns? (investigating structural attributions)
7. What visions for human-AI futures emerge? (identifying prescriptive themes)

Iterative coding process: The initial coding remained close to the data, utilizing participants’ language where possible. We coded for semantic and latent meaning, capturing both explicit content and underlying assumptions. Second-cycle coding consolidated initial codes into candidate themes through a process of constant comparison. We sought patterns across the dataset while remaining attentive to divergent cases.

Theme development: Themes were constructed through iterative refinement, ensuring internal homogeneity and external heterogeneity. We consolidated the initial 10 themes into seven final themes by combining conceptually related patterns. For instance, “AI as Corporate Justification” merged with “Shattered Psychological Contract” as both addressed trust and betrayal. Each theme required substantial data support (minimum 20 comments) with clear conceptual boundaries.

### 3.5 Integration strategy

Mixed-methods integration occurred at multiple points. Quantitative findings informed qualitative sampling. Comments were purposively selected to show sentiment disagreement between VADER and BERT for in-depth analysis. Topic models provided initial thematic categories, which were refined through qualitative coding. Network clusters guided attention to interconnected concepts during the interpretation process.

The integration philosophy followed complementarity logic; each method addresses the limitations of the others. Computational breadth complements qualitative depth; pattern identification supports meaning interpretation; statistical regularities contextualize individual narratives. This multi-level integration produces findings neither method could achieve independently.

### 3.6 Ethical considerations

#### 3.6.1 Studies involving human subjects

This study involved secondary analysis of publicly available Reddit comments. The researcher holds active ethical clearance from the University of Johannesburg’s Department of Industrial Psychology and People Management Research Ethics Committee (IPPM-2022-618(D), valid until 12 June 2026) for the broader doctoral research program. However, this specific component analyzing publicly available Reddit data does not constitute human subjects research requiring formal ethics review under University of Johannesburg policies.

According to the University of Johannesburg’s adopted guidelines (Department of Health, Republic of South Africa, 2015), “*Research that relies exclusively on publicly available information or accessible through legislation or regulation usually need not undergo formal ethics review*” (Section 1.1.8, p. 9). The guidelines further specify that research involving observation of people in public spaces (including digital spaces) or secondary use of anonymous information is typically exempt from formal review when specific criteria are met: (1) no direct interaction with individuals or groups, (2) no staged intervention, (3) individuals have no reasonable expectation of privacy, (4) findings do not identify individuals or groups, and (5) no identifiable information is generated during the research process (Section 4.3.2, p. 34).

This research meets all exemption criteria specified in the University of Johannesburg’s ethics framework:

Publicly available information: The data consists entirely of comments posted on Reddit’s r/AskReddit forum, a public platform where content is freely accessible without authentication requirements. Reddit’s terms of service and platform design make explicit that user contributions are publicly viewable and may be accessed by third parties (Reddit, 2025).

No direct interaction or intervention: The research involved no recruitment, contact with, or intervention involving participants. All data was accessed retrospectively in compliance with platform terms of service. No interaction with individual users occurred.

No reasonable expectation of privacy: Reddit users posting in public forums operate under contextual norms of publicity. The platform architecture, cultural practices, and terms of service establish that r/AskReddit posts are intended for broad public consumption (Gliniecka, 2023; Proferes et al., 2021).

No identification of individuals: All usernames and personal identifiers were excluded from data collection, analysis, and reporting. Findings are presented in aggregate form or with numerical participant identifiers (e.g., Participant 1). Verbatim quotes are contextualized to prevent reverse-identification through search engines (Adams, 2024; Reagle, 2022).

No generation of identifiable information: The research process generated no new identifiable information about participants. Analysis focused on textual patterns, themes, and discourse structure rather than individual attribution.

While formal ethics review was not required under institutional policy, we nevertheless adhered to established ethical principles for social media research (Fiesler et al., 2024; Markham and Buchanan, 2012; Proferes et al., 2021). We applied “situated ethics” recognizing that even public data about sensitive topics (job loss, mental health) warrants protective measures. Our approach prioritized participant protection despite the exemption status, implementing de-identification protocols and minimizing potential risks through careful data handling and presentation.

#### 3.6.2 Inclusion of identifiable human data

No potentially identifiable images or data are presented in this study. All participant references use numerical identifiers. Direct quotations are limited and presented without user attribution.

## 4 Findings

### 4.1 Quantitative patterns: mapping the emotional and semantic landscape

The computational analysis revealed a complex emotional and thematic landscape characterized by profound ambivalence and interconnected concerns about work, identity, and human value in an algorithmic age.

Sentiment divergence and emotional complexity: The stark disagreement between sentiment analysis methods illuminates the emotional complexity of AI discourse. VADER’s lexicon-based approach classified 52.2% of comments as positive, 32.5% as negative, and 15.3% as neutral. In striking contrast, the contextual BERT model identified 51.1% as negative, 37.0% as neutral, and only 11.9% as positive. This reversal (from majority positive to majority negative) represents more than methodological variance; it reveals how workers employ linguistic strategies to cope with distressing experiences.

Manual analysis of the 659 comments where models disagreed most strongly (VADER positive, BERT negative) uncovered consistent patterns of ironic positivity and resigned acceptance. Comments like “Great news, I’m free from that soul-crushing job thanks to our AI overlords” exemplify this pattern; surface markers of positivity (“great,” “free”) mask deep negativity captured by contextual analysis. This linguistic strategy serves multiple functions: maintaining face while expressing distress, using humor to process trauma, and performing resilience while experiencing vulnerability.

Emotional ambivalence and co-occurrence: The NRC emotion analysis revealed striking emotional co-occurrence patterns. Trust words appeared in 13% of comments, but closer examination showed that these often expressed broken trust (“trusted my employer,” “cannot trust companies anymore”). Fear (6%) and sadness (5%) co-occurred in 67% of cases, suggesting compound negative emotional states. Anticipation (9%) appeared split between positive (excitement about new opportunities) and negative (dread about future automation) valences.

Anger (4%) concentrated in comments about corporate behavior rather than technology itself. Workers directed anger at “greedy executives,” “shareholder capitalism,” and “consultants who have never done real work.” This attribution pattern suggests workers blame human decisions about AI implementation rather than technology itself—a critical distinction for intervention design.

Thematic architecture: Topic modeling revealed eight statistically distinct themes with clear interpretive meaning:

Topic 1 (12.3%): “Adaptation and Learning” - Featured terms like “learn,” “new,” “skills,” “adapt,” suggesting active responses to AI challenges.

Topic 2 (20.6%): “Corporate Power and Workplace Politics” - Dominated by “company,” “management,” “shareholders,” “profits,” indicating structural critiques.

Topic 3 (9.7%): “Technical Realities” - Included “algorithm,” “data,” “model,” “error,” showing technical literacy and system critique.

Topic 4 (11.2%): “Emotional Processing” - Centered on “feel,” “scared,” “anxious,” “depressed,” revealing psychological impacts.

Topic 5 (11.4%): “Resistance and Critique” - Featured “refuse,” “fight,” “wrong,” “human,” expressing active opposition.

Topic 6 (7.9%): “Economic Impacts” - Focused on “salary,” “bills,” “unemployment,” “savings,” addressing material consequences.

Topic 7 (17.9%): “Job Loss and Replacement” - Dominated by “replaced,” “fired,” “automated,” “obsolete,” capturing displacement experiences.

Topic 8 (10.8%): “Future Uncertainty” - Included “future,” “career,” “years,” “survive,” expressing temporal anxiety.

Keyword Distinctiveness: TF-IDF analysis confirmed the centrality of the human-AI-work intersection. Beyond expected terms (“AI,” “job,” “work”) distinctive keywords revealed specific concerns. “Copilot” appeared 47 times indicating the widespread impact of GitHub’s coding assistant. “Creative” ranked surprisingly high (TF-IDF score: 0.73) challenging assumptions that AI primarily threatens routine work. “Betrayed” scored higher than “unemployed,” suggesting psychological impacts outweigh economic concerns for many workers.

Network structure and community formation: The co-occurrence network revealed three tightly interconnected communities with bridge terms facilitating cross-cluster communication:

Community 1 (Employment/Automation): Core terms included “job,” “work,” “replaced,” “company,” “AI,” forming the network’s gravitational center. This cluster’s centrality (average degree: 24.3) indicates employment concerns anchor all discourse.

Community 2 (Ethics/Emotion): Centered on “human,” “feel,” “wrong,” “trust,” “fair,” this cluster connected emotional and moral dimensions. “Human” served as the primary bridge term, appearing in 73% of edges between communities.

Community 3 (Technical/Systemic): Focused on “system,” “algorithm,” “data,” “technology,” “error,” representing technical literacy and systematic critique. This smaller cluster (18% of nodes) showed surprising sophistication in technical understanding.

The network’s high clustering coefficient (0.67) indicates dense local connections within communities, while moderate average path length (2.4) shows efficient global communication across topics. This structure suggests integrated rather than fragmented discourse—workers simultaneously process practical, emotional, and ethical dimensions.

### 4.2 Qualitative themes: lived experiences of algorithmic disruption

Seven major themes emerged from thematic analysis, each representing distinct yet interconnected dimensions of algorithmic anxiety:

#### 4.2.1 Theme 1: shattered trust and corporate betrayal

Participants described profound betrayal when organizations replaced human teams with AI. A data scientist (Participant 1, 10,861 upvotes) captured this violation: *“I used to be a data scientist (with 13 years of experience). My boss wanted me to solve a problem which involved clustering sensor data by location. Because errors in latitude and longitude tend to be random, we’ll have elliptical clouds of points, so I said we should use k-means. My boss picked up his laptop, turned it around, and said: ‘But Copilot says that we should use DBSCAN. I researched DBSCAN and found that it would be very slow and do the wrong thing in a worse way. My boss did not agree. I was laid off a few weeks later, along with the rest of the data team.”*

The Microsoft vendor engineer (Participant 12, 1,111 upvotes) described being forced to train their AI replacement: *“We are just expected to work as normal and keep ‘training’ this AI until our last day at the end of the year. It’s malding and insanity.”* This experience of training one’s replacement while being strung along with false promises exemplified the breach of the psychological contract. As Participant 8 (1,641 upvotes) observed: *“It’s like the people in charge of companies would really prefer not to have employees at all. Businesses boast about their ‘revenue per employee’ metrics. American-style management does not want to have employees.”*

A few more relevant comments:

- It is every CEO’s w*t dream to replace humans with A. I.
- *Companies tell you it’s “AI,” but it’s just an excuse to cut staff.*
- *A $20 k license for software that’s ‘good enough is better than a team of people making great work product at $300 k + fringe, so sayeth the Wall Street Gods of Old.*
- *Like when computers and productivity software got popular, secretaries did not lose their jobs. Companies just stopped hiring secretaries.*
- *Keep in mind, most people will not directly lose their jobs to AI. Companies will just hire fewer people and expect the current people to get more done.*

#### 4.2.2 Theme 2: identity and meaning erosion

Professional identity emerged as a casualty of AI integration, when workers found their core tasks redefined or devalued. For many creative professionals, AI tools were introduced as “assistants” but rapidly shifted the fundamental nature of their work from original creation to simple editing (curation and correction). A graphic designer (Participant 2, 5,565 upvotes) whose firm adopted AI image generation tools within a six-month period articulated this transformation*: “The job is becoming less about executing the first idea and more about curating, refining, and adding the crucial human touch (and catching AI’s weird mistakes). It feels less like I lost my job and more like my job description was rewritten overnight.”* This participant described moving from conceptualizing and creating original designs to primarily reviewing and correcting AI-generated options, a shift that eliminated the creative process they had spent years developing.

Some responded through career pivots seeking meaning. These transitions typically involved leaving knowledge work entirely for fields perceived as more resistant to automation or offering clearer human value. Participant 24 (436 upvotes) stated: *“I’m shifting to social work to hopefully have a more meaningful career.”* This participant had worked in payroll administration before their role was automated, and explicitly framed the career change as seeking work where “helping real people” provided intrinsic meaning that algorithmic efficiency could not replicate. The literary editor (Participant 3, 4,690 upvotes) captured the irony: *“So after spending 15 years working for the greater good of sci-fi, I got outsourced to a goddamn robot. To be fair, I guess I probably should have seen that coming, given the genre.”* This participant’s publisher had replaced human editorial review with AI text analysis tools that assessed manuscript marketability.

A few more relevant comments:

- *I was set to work on a children’s book for the school I worked for. I told them straight away I would not sign up if there was AI (they used AI art for their last children’s book, and apparently the kids hated it). They laughed and told me it was all going to be from my imagination. So I wrote it and started to put together some clip art, as they had asked me to make the book. Then I had a lupus flare-up. I was out for a whole week, and when I came back, they said the book was ready. However, when I looked at it, yes, it was my writing, but all the clip art had been replaced with the ugliest AI art I had ever seen. I faked ignorance and asked who illustrated it. Excitedly, my boss showed me the AI tool she used. I cried all the way home and for the next few hours. I was already a fiction author and if this new book with AI art got onto Amazon like they planned, it was going to ruin my reputation as a very anti-AI author. I texted and asked to have my name removed. One batch had already been sent out, and it was too late, but they felt bad and decided to give me a pen name for the next few batches. I parted ways soon after that.*
- *Worked payroll, then got replaced, now I work food service again, and the existential angst about money and my career trajectory hits harder in my 30s than 20s. I’m so lucky to have my fiancé be so supportive, at least. Been applying to payroll jobs ever since, gotten a few interviews, but no satisfactory offers*.

#### 4.2.3 Theme 3: technostress and coerced adoption

Mandatory AI integration created intense strain. The Microsoft vendor engineer (Participant 12, 1,111 upvotes) described coercion: *“This AI became a mandatory metric where we could get fired for not using it. This AI was and is almost always wrong with technical information and always wrong on key details when assessing complex issues. Essentially it was completely useless if you have any semblance of competency in your role.”*

The graphic designer (Participant 2, 5,565 upvotes) added: *“The pressure to constantly adapt is the real challenge.”* Participant 19 (692 upvotes) expressed FOMO-driven stress: *“I feel I am falling behind as most people are actively using AI in their creative careers which I do not because you need to buy credits to do anything, I do not wanna be chained.”*

A few more relevant comments:

- *This bet on “AI” originating from the US-based companies is so weird to me. It seems to be so detrimental, yet most of the leadership seems to be so committed, it feels almost like a cult. As somewhat of a bystander, it feels like China’s bet on electrification and exports of technology related to sustainable energy seems so much better, and yet, US folks seem to be doubling down on their initial stance.*
- *All jobs are (indirectly) affected. I’ve seen people say, “Oh, well, I’m a chef, and that will take much longer to automate.” Everyone who’s been laid off by AI will want these few remaining jobs now, so your competition is increasing exponentially. The odds of you becoming a chef, or retaining your chef job, will decrease drastically, even if the job itself remains manual labor for a while longer.*
- *They just started introducing more AI things to “save us time so we can focus on the important stuff,” and they swore they were not trying to replace people; they just wanted to help us at work. Then they eliminated positions one by one. Once they had eliminated 2 jobs and moved the responsibility to my role (so the work of 3 positions), they started laying off those people.*

#### 4.2.4 Theme 4: devaluation of expertise

Experienced professionals described humiliation when algorithms overruled judgment. The literary editor (Participant 3, 4,690 upvotes) expressed: *“After spending 15 years working for the greater good of sci-fi, I got outsourced to a goddamn robot… Just never thought it’d surpass human reading/analysis THAT fast.”* Though Participant 10 (1,306 upvotes) countered: *“It has not surpassed human reading and analysis. Your company is just soulless and greedy.”*

Participant 14 (901 upvotes) warned about systemic consequences: *“They’ll have fired all their experts and replaced them with a computer who knows how to interview well but in practical terms is fresh out of college.”* A designer (Participant 9, 1,566 upvotes) shared: *“My team and I spent a few days working on a branding… Then our boss AI-generated a (very crappy) mood-board + logo, and presented it to the client then shoved it in our faces like ‘haha see should’ve used AI from the start, the client loved it’.”*

A few more relevant comments:

- *I have been saying this since its inception. An increasingly common observation is that AI amplifies the Dunning-Kruger effect. It gives the layman a very convincing delusion of competency while actually being confidently and objectively wrong in both nuanced and obvious ways.*
- *I teach at university and this describes my experience with my students using AI to a T. They’ll have a very general idea of the material, which we want them to deepen by doing some sort of project or essay or the like, and they’ll offload it to ChatGPT, polish the results a bit and get rid of the em dashes and then act super surprised when we tell them it’s shitty work. Because like… If you have a very general idea of what you are talking about, it sounds perfectly plausible.*
- *I’m an epidemiologist working for a local health department in a team building disease surveillance capacity. Basically, my team makes data cleaning and visualizations automated so we can spend time interpreting the output and detecting outbreaks and patterns earlier. We all have master’s or PhDs in epidemiology/biostatistics. We are being pushed out in the middle of respiratory season at the end of the year, so the IT team can make oversimplified graphs that are not useful and use AI for the rest. It’s absolutely horrifying that our community’s health is in the hands of untrained IT and AI.*
- *This seems to be the big appeal of AI for bosses. It allows people who do not know how to do things to* look *like they can do things. So yeah, your boss is like “look at me, I’m a graphic designer/data scientist/whatever” when they have no idea what you actually do or how to evaluate the AI results.*
- *We had one client do their original concepts with AI, which is fine for them to communicate what they need to a designer. But they got so attached to this initial concept, they did not want to pay a designer to recreate it so we could actually use it for print. They did not see the mistakes. They did not understand the basic concept that trying to put something that’s 10”x10” on a flat screen is not going to be usable for what we need, printing and installing these graphics on a truck, a 3D object. Or logo creation, sure, you can use it for small print, but not large unless you have a vector file. AI cannot vectorize well yet, especially if they got gradients and effects all over it. People are starting to do everything and anything to not pay a designer, even if it takes up more of their time.*

#### 4.2.5 Theme 5: job insecurity and future anxiety

Existential uncertainty permeated discussions. Participant 47 (203 upvotes) worried: *“My teams in Uruguay and China were really good, but maybe even those guys are at risk of loss of work to an AI. Frightening.”* Participant 55 (151 upvotes) expressed paralysis: *“Every time I consider pivoting to a new career (I’m unemployed), I have massive doubts it’s even going to exist in this cluster fuck of a time.”*

Participant 85 (90 upvotes) explained systemic drivers: *“CEOs are legally obligated to invest in AI as a business strategy, as it props their stock up today, and is marketed as a requirement for long-term success.”* Participant 74 (102 upvotes) predicted broader impacts: *“My hypothesis is that we are about to see a drop off in expertise and specialization.”*

A few more relevant comments:

- *Until they make robots that can work in a kitchen, my job as a chef is safe. I give it 10 years.*
- *I’m a teenager and just seeing how bad the AI stuff is getting is really scaring me and making me lose hope*: *(… I wanted to be a scientist, but I’m not sure if that’ll even be an option for me)*
- *I work a bunch of different positions in audio post production, and it’s only a matter of before a director or producer tells me that the talent has agreed to let AI clone/train their voice so I can do edits and pick-ups without having to re-record them. So far, it has not been a thing, but the clock is ticking. Ultimately, it means less money for me and less money for the talent, so I’m holding off for as long as I can.*
- *My uncle does voice acting. A lot of his work came from audiobooks, but that’s drying up lately and being replaced by AI voice.*
- *Graphic designer here. I have not been fully replaced yet, but the landscape has completely changed. Clients now expect me to use AI as a ‘co-pilot’, generating initial concepts, mood boards, and even rough copy in minutes, not hours. The job is becoming less about executing the first idea and more about curating, refining, and adding the crucial human touch (and catching AI’s weird mistakes). It feels less like I lost my job and more like my job description was rewritten overnight. The pressure to constantly adapt is the real challenge.*
- *I was a full-time visual artist. The commissions dried up when people started using ChatGPT to make all their images, flyers, posters, etc.*
- *I’m now an OF content creator… after losing a marketing copywriter job.*

#### 4.2.6 Theme 6: cynical adaptation and quiet resistance

Strategic disengagement emerged as a coping mechanism. Participant 220 (18 upvotes) advised: *“Stop pushing to improve your output, just press the button on the robot. Stop innovating, just press the button on the robot. Stop trying to impress and showcase your ability, just press the button on the robot. Keep your creativity for yourself. You’ll be paid or laid off either way.”*

Others adapted pragmatically. Participant 698 (2 upvotes) shared: *“I realized AI is inevitable, so now I’m in school as a tradesman looking to be a welder.”* Participant 694 (2 upvotes) described the new reality: *“customer-facing support roles have become a nightmare due to AI. customer-facing jobs that were once plentiful are few and far between, and absolutely misery.”*

#### 4.2.7 Theme 7: human touch as enduring value

Despite pessimism, participants affirmed uniquely human qualities. A voice actor (Participant 4, 3,770 upvotes) described principled resistance: *“I’ve worked on campaigns where all of the voice artists refused to sign their contract because there’s a new clause in it - if they sign it, they are signing the rights of their voice over to AI… They all refused; the client just recast all of them.”*

Participant 694 (2 upvotes) noted customer preferences: *“customers still crave human interaction, especially for technical support or support scheduling.”* The graphic designer (Participant 2, 5,565 upvotes) emphasized “adding the crucial human touch” as remaining valuable, while acknowledging the transformed nature of their work.

Another relevant comment:

- *I have not lost my job, and I doubt I will, but I find the way people are using AI* like *my job to be extremely concerning. I’m licensed on a national and state level as a mental health therapist. Right now, I do evaluations for mental health services and am not providing direct therapy services, but regardless, I do not think AI is capable of either of those jobs (at least, not yet). There is a certain level of empathy you have to have to do the job(s), and I’m not sure a machine can fake it well enough. In theory, I do not think evaluations could be replaced by AI, assuming clients could type back answers, but most people I see are woefully technologically illiterate, if not outright Luddite/technophobic and refuse to interact with computers because they do not know how. I think even providing voice responses to a machine or an AI-avatar would be so off-putting. People bitch enough now about doing telehealth, which has become very widespread post-COVID. All that to say, the trend or ‘replacing’ a therapist with AI is something that disappoints, frustrates, and outright scares me.*
- *What I do not understand is how people can prefer AI VAs over actual artists. Take a look at any video with AI-slop voice over, and regardless of the content, that’s what the comments will be about*.

## 5 Discussion

### 5.1 Theoretical contributions and implications

Our findings extend existing theoretical frameworks while also revealing their limitations in explaining the impacts of AI in the workplace. The integration of multiple theories proves essential, as no single framework can capture the full complexity of algorithmic anxiety.

### 5.2 Algorithmic anxiety: an integrative framework

While the term “algorithmic anxiety” appears in scholarship examining the paradoxical relationship between technological promises of control and the widespread fears algorithms unleash across domains including surveillance, identity, and the outsourcing of human decision-making to smart machines (De Vries and Schinkel, 2019; Elliott, 2024), its specific manifestation in employment relationships (where AI mediates decisions about livelihood, professional worth, and economic security) remains undertheorized. Existing constructs address discrete elements—technostress captures technology-use strain (Tarafdar et al., 2019), job insecurity addresses employment fears (Shoss, 2017), and automation anxiety focuses on replacement concerns (Brougham and Haar, 2018). However, our empirical analysis reveals that workers experience not discrete, separable anxieties but a compound psychological phenomenon integrating multiple dimensions simultaneously.

We position algorithmic anxiety as an umbrella construct encompassing seven interrelated dimensions identified in our thematic analysis. Shattered trust captures the experience of corporate betrayal when organizations frame AI as “assistive” while using it to eliminate positions or devalue expertise—a psychological contract breach unique to being deceived about technology’s true purpose. Identity erosion reflects the loss of professional self-concept when core competencies become automated, leaving workers to curate algorithmic outputs rather than exercise craft. Technostress and coerced adoption describe the strain of being forced to use and train systems that threaten one’s role, creating the psychologically contradictory position of facilitating one’s own potential obsolescence. Expertise devaluation captures the deflation experienced when skills developed over years become worthless overnight, not through personal failure but through algorithmic advancement.

Future anxiety extends beyond current job loss to encompass existential uncertainty even among currently employed workers who recognize their potential future in others’ present displacement. This anticipatory dimension distinguishes algorithmic anxiety from traditional job insecurity—workers fear not just losing this job but the possibility that no human expertise retains lasting value. Cynical adaptation represents defensive coping through dark humor, resignation, and quiet resistance—attempts to maintain psychological equilibrium while acknowledging limited agency. Finally, human value affirmation reflects efforts to assert inherent worth beyond productivity, countering the implicit message that human work has become redundant.

These seven dimensions constitute algorithmic anxiety as a compound phenomenon distinct from existing constructs in three ways. First, it integrates cognitive (uncertainty about the future), affective (betrayal, deflation), and behavioral (resistance, adaptation) responses within a single framework. Second, it captures both actualized distress (among displaced workers) and anticipatory distress (among those witnessing displacement), explaining why narratives of job loss receive widespread validation from workers not yet affected. Third, it addresses the unique circumstance of being evaluated, managed, and potentially replaced by systems one helped create, introducing dimensions of betrayal and complicity absent from traditional automation frameworks where workers and machines occupied clearly separate spheres.

Our contribution is not introducing a term but empirically characterizing algorithmic anxiety’s constituent dimensions through workers’ own accounts. Future research should develop measurement instruments capturing these seven dimensions, examine whether they emerge as distinct factors or load onto higher-order constructs, and assess whether algorithmic anxiety predicts unique variance in outcomes beyond what established constructs explain individually.

Extending psychological contract theory: Traditional psychological contract theory assumes human agents on both sides of the employment relationship. Our findings reveal how AI fundamentally disrupts this assumption, introducing what we term “algorithmic mediation” of psychological contracts. Workers experience AI not as neutral tools but as quasi-agents making decisions previously reserved for human managers. This creates novel breach types: “technological betrayal” (when AI systems workers helped build and replace them), “algorithmic abandonment” (when human judgment is systematically devalued), and “digital dehumanization” (when workers become data points for algorithmic processing).

The temporal dimension proves critical. Traditional contract breaches occur at discrete moments, such as a broken promise or a layoff announcement. AI-mediated breaches unfold gradually through incremental automation, creating “breach cascades” where each small violation compounds the effects of previous ones. Workers describe a “thousand cuts” phenomenon where no single change seems breach-worthy, but cumulative impact devastates trust.

Reconceptualizing Technostress: Our findings suggest existing technostress frameworks inadequately capture AI-specific stressors. We propose “algorithmic technostress” as a distinct construct with unique characteristics. Unlike traditional IT stress, which focuses on tool usage, algorithmic stress involves existential dimensions, questioning human value, purpose, and the future viability of humanity. The stressor is not technology itself but technology’s implications for human worth.

We identify novel stress mechanisms: “performance anxiety loops” (perpetual evaluation by opaque systems), “competence inversion stress” (when expertise becomes liability), and “automation anticipation stress” (chronic anxiety about future displacement). These mechanisms operate simultaneously, creating compound stress that exceeds the sum of its parts.

Self-determination theory in algorithmic contexts: AI systematically undermines all three basic psychological needs, but through mechanisms SDT does not fully anticipate. Autonomy loss occurs not just through external control but through “algorithmic channeling,” where AI shapes decision spaces so fundamentally that genuine choice becomes impossible. Competence threats extend beyond current performance to “prospective competence anxiety,” doubt about any skill’s future relevance. Relatedness suffers through “algorithmic intermediation,” where human connections become filtered through AI systems.

Conservation of resources in accelerated change: COR theory helps explain cascading impacts, but AI’s pace challenges the framework’s assumptions about resource cycles. Traditional resource loss and gain occur over predictable timeframes. AI creates “resource volatility” where valuable skills become obsolete overnight, while new requirements emerge faster than acquisition possibilities. This creates perpetual resource-deficient states, where workers cannot build resources quickly enough to offset losses.

### 5.3 Digital discourse as collective sense-making

Reddit discussions represent more than individual venting; they constitute collective sense-making about technological transformation. Through upvoting, commenting, and sharing experiences, workers collaboratively construct narratives about the meaning of AI and appropriate responses. This process serves multiple functions: emotional validation, sharing practical strategies, and ideological resistance to corporate AI narratives.

The platform’s affordances (pseudonymity and community validation) create unique conditions for authentic disclosure and collective processing. Workers who share experiences too threatening for workplace expression receive validation from others with similar experiences and access accumulated wisdom about coping strategies. The discourse becomes a parallel institution to formal workplace structures, providing support and resistance that corporate environments deny.

## 6 Implications for practice and policy

The following implications derive from workers who experienced AI-driven workplace transformation negatively, within predominantly Western contexts, at a specific moment during AI’s rapid evolution. Given our sampling approach and data limitations, these recommendations should not be interpreted as universally applicable across all cultural contexts, organizational types, or AI implementation scenarios. Rather, they identify risk factors, warning signs, and protective strategies relevant to preventing or mitigating the negative experiences documented in our findings. Organizations in non-Western contexts, those implementing AI successfully, or those operating in different cultural environments may require adaptations of these principles. These implications represent hypothesis-generating insights requiring validation through comparative research across diverse contexts and longitudinal studies tracking implementation outcomes over time. They are offered as initial guidance for practitioners navigating AI implementation, recognizing that successful approaches likely vary by institutional context, cultural norms, and temporal factors.

Our findings suggest fundamental reconsideration of AI implementation approaches. Current strategies prioritize technical optimization while treating human impacts as secondary “change management” concerns. This approach generates the resistance, cynicism, and disengagement that our data documents. While our findings derive from Western-centric contexts where workers experienced AI negatively, they suggest that alternative approaches centering human experience from inception may mitigate these risks.

Participatory AI governance: In contexts similar to those documented in our study—Western, knowledge-work environments undergoing AI-driven transformation—workers must participate meaningfully in AI adoption decisions, not through token consultation but genuine co-design. This includes representation in selection processes, implementation planning, and ongoing evaluation. Our data shows that workers possess a sophisticated understanding of AI’s capabilities and limitations; excluding this knowledge impoverishes implementation and generates resentment. However, the form such participation takes may differ across cultural contexts, particularly in collectivist societies where representation mechanisms operate differently than in individualist Western settings.

Transparent communication: Organizations must honestly communicate AI’s intended role and impact. Our findings reveal that workers particularly resent deception, being told AI will “augment” work while planning replacement. Transparency about automation plans, even when difficult, generates less betrayal than discovered deception.

Meaningful reskilling: Current “reskilling” initiatives often offer superficial training in using AI tools rather than developing AI-complementary capabilities. Workers need pathways to genuinely secure roles, not temporary reprieves before the next wave of automation. Effective programs require strategic design that addresses AI’s rapid evolution. Siemens’ digital learning platform demonstrates key success factors: providing personalized learning paths based on individual skill gaps, offering microlearning modules that fit within work schedules, and creating clear connections between training and actual job requirements (Freise et al., 2025). Critically, Siemens invested in infrastructure that made learning accessible during work hours rather than expecting employees to reskill in their personal time while facing job insecurity.

However, our findings suggest workers remain skeptical of employer-sponsored reskilling when organizations simultaneously automate positions, perceiving programs as symbolic gestures rather than genuine commitments. Reskilling alone cannot address structural displacement when entire occupational categories face automation. Organizations must honestly assess whether programs provide genuine security or merely delay inevitable displacement, and provide appropriate transition support accordingly.

Ethical frameworks: Organizations (particularly in Western contexts where our data was generated) need explicit ethical frameworks governing the role of AI in the workplace. These must address not just bias and privacy but human dignity, meaningful work, and fair transition support for displaced workers. Our findings suggest workers judge organizations more on how they implement AI than whether they implement it. The specific content of such frameworks will necessarily vary across cultural and institutional contexts, as notions of dignity, meaningful work, and fair treatment are culturally mediated.

### 6.1 Societal implications

This study documents patterns within Western digital discourse about AI-driven job displacement that suggest broader societal challenges, though the specific manifestation of these challenges may vary across cultural and economic contexts. Individual organizational efforts, however well-intentioned, cannot address systemic issues such as technological unemployment, meaning crises in automated economies, or the value of human beings in an age of artificial general intelligence.

Policy interventions in contexts similar to those represented in our data (Western liberal market economies) might include stronger worker protections during technological transitions, requirements for human oversight in algorithmic decision-making, public investment in human-centric sectors that are resistant to automation, and the evolution of the social safety net to acknowledge automation’s impact. Some participants mentioned a universal basic income, although views remained divided on whether this addresses concerns about meaning and purpose beyond economic security. Policy responses in other institutional contexts (such as coordinated market economies with strong labor protections, or developing economies with limited social safety nets) would require different configurations balancing worker protection with economic development goals.

Our findings underscore the importance of proactive workforce preparation for AI integration. Research on psychological resilience in future workplaces demonstrates that organizations investing in protective factors (psychological safety, participatory decision-making, supportive leadership) before implementing AI can prevent the algorithmic anxiety our study documents (Alitabar and Parsakia, 2025). Foresight methodologies involving workers in scenario planning may help them develop adaptive capacity before encountering displacement threats. This suggests treating workforce resilience as infrastructure requiring advance investment rather than post-crisis damage control.

Educational systems require fundamental reconception. Current approaches emphasize technical skills that are increasingly automated, while neglecting uniquely human capabilities such as ethical reasoning, emotional intelligence, and creative problem-solving. Career counselling must acknowledge radical uncertainty, preparing students not for specific careers but for the ability to adapt. These educational recommendations are framed within Western educational paradigms; their applicability to other educational systems and cultural contexts requires careful consideration of local institutional arrangements and cultural values regarding education, work, and human development.

## 7 Limitations and future directions

Several limitations constrain the generalizability and interpretation of our findings. Reddit’s user base skews younger, more technologically literate, male, and Western-centric than the general working population (Proferes et al., 2021). More critically, the thread prompt *(“Hey people who lost their jobs to AI, what happened?”)* creates selection bias toward negative experiences, attracting workers who experienced AI as threatening while excluding those with neutral or positive experiences. Community engagement through upvotes suggests the discourse resonates with workers experiencing anticipatory rather than actualized displacement, but we cannot verify the actual experiences of those engaging with the content. Pseudonymity prevents demographic verification or systematic analysis of participant characteristics.

These sampling characteristics have important interpretive consequences. Our thematic findings (shattered trust, identity erosion, technostress) describe psychological responses among workers negatively affected by AI, not universal reactions to workplace AI. The 51% negative sentiment in our corpus cannot be extrapolated to estimate how commonly workers experience AI negatively in general populations. Our theoretical contributions illuminate mechanisms operating among distressed workers rather than invariant laws applying to all AI contexts. Claims about the prevalence, frequency, or inevitability of algorithmic anxiety would be unwarranted given our sampling approach. Our findings characterize the phenomenology of negative AI impact, providing depth of understanding about this experience while requiring complementary research on representative samples to establish its prevalence.

Our cross-sectional design captures a temporal snapshot during the rapid evolution of AI. Longitudinal research could track how worker attitudes evolve as AI capabilities expand and societies adapt, specifically examining whether initial algorithmic anxiety diminishes with familiarization, whether predicted job losses materialize at anticipated rates, and how coping strategies shift over time. Panel studies following the same workers across multiple years of AI implementation would reveal whether psychological contract breaches prove temporary disruptions or permanent shifts in employment relationships.

Comparative studies across cultures with different labor protections, technological attitudes, and social contracts would illuminate contextual factors shaping AI’s impact. Specific research questions merit investigation: Do workers in Nordic countries with strong social safety nets experience less algorithmic anxiety than those in liberal market economies? How do collectivist cultures (where job loss affects family honor and social standing) differ from individualist cultures in processing AI displacement? Do nations with codetermination rights (requiring worker consultation in technology decisions) show different implementation outcomes than those without such protections? Cross-national comparisons could identify which institutional features mitigate negative impacts and which cultural factors predict resistance versus acceptance.

Future research should examine positive cases, organizations successfully integrating AI while maintaining worker well-being and dignity. What differentiates these contexts? How do workers experience AI as genuinely augmenting rather than replacing? Understanding success conditions is essential for practical guidance.

Methodologically, our approach demonstrates mixed-methods value for complex sociotechnical phenomena. Future studies could extend this integration, perhaps combining digital discourse analysis with workplace ethnography and physiological stress measures. Real-time data collection during AI implementation can capture the evolution of worker responses, rather than relying on retrospective accounts.

## 8 Conclusion

This study illuminates the human dimensions of workplace AI integration through analysis of digital discourse from those directly experiencing algorithmic disruption. Our mixed-methods approach reveals algorithmic anxiety as a complex syndrome encompassing not just job insecurity but fundamental threats to identity, meaning, and human value in increasingly automated workplaces.

The sentiment divergence between surface positivity and contextual negativity suggests workers employ sophisticated coping strategies (humor, irony, resignation) while experiencing genuine distress. The seven themes emerging from thematic analysis paint a picture of profound transformation where traditional psychological contracts shatter, professional identities erode, and workers struggle to maintain dignity and purpose as machines assume previously human roles.

Theoretical contributions include extending psychological contract theory to accommodate algorithmic mediation, identifying AI-specific technostress mechanisms, demonstrating systematic undermining of basic psychological needs, and revealing cascading resource loss in accelerated technological change. These frameworks require further development to fully capture AI’s novel challenges to established organizational theories.

The digital discourse analyzed represents more than individual grievances, it constitutes collective sense-making about one of the most significant transformations in work history. Through Reddit’s pseudonymous platform, workers create parallel institutions for processing experiences, sharing strategies, and constructing counter-narratives to corporate AI rhetoric. This grassroots response warrants attention from scholars and practitioners seeking to understand the true impact of AI.

Practical implications emphasize that sustainable AI integration requires fundamental reconsideration of implementation approaches. Technical optimization without human consideration generates the resistance and cynicism our data documents. Organizations must genuinely involve workers in AI governance, communicate transparently about automation plans, provide meaningful reskilling opportunities for secure roles, and establish ethical frameworks that protect human dignity.

The path forward requires recognizing AI integration as a fundamentally human challenge, not a technical problem. Success metrics must expand beyond efficiency gains to include worker well-being, organizational trust, and societal flourishing. This demands new forms of human-AI collaboration that preserve what makes work meaningful while leveraging AI’s capabilities.

As we stand at this historical inflexion point, choices made about AI’s workplace role will reverberate for generations. Our findings suggest current approaches often fail to account for human costs, generating unnecessary suffering while undermining potential benefits. Alternative paths exist, ones that center human dignity, preserve meaningful work, and create genuinely augmented rather than diminished human potential.

The workers whose voices animate this study offer both warning and wisdom. They warn of futures where humans become secondary to systems they created, where expertise becomes obsolete overnight, where meaning drains from work reduced to algorithmic supervision. But they also affirm enduring human qualities (creativity, empathy, ethical judgment, relationship) that no algorithm replicates.

The ultimate measure of our technological progress will not be the sophistication of artificial intelligence, but rather the wisdom in its integration with human life. This study contributes to that wisdom by amplifying voices from the front lines of automation, translating their experiences into theoretical insights and practical guidance. Their message deserves to be heard: preserve the human in human-AI collaboration, or risk losing not just jobs but the meaning, dignity, and purpose that make work fundamentally human.

## Data Availability

The computational analysis code, topic modelling outputs, network analysis files, and statistical results are available from the corresponding author upon reasonable request. Researchers may access the restricted data under a restricted data use agreement that requires institutional ethics approval and adherence to the protective protocols described in Section 3.6.
